# Initial evidence of effects of a novel digital behavioural treatment for chronic pain: A series of replicated randomized single-case experimental design studies

**DOI:** 10.1177/20552076261467865

**Published:** 2026-07-14

**Authors:** Haya Al Sharaa, Sara Laureen Bartels, Patrick Onghena, Afra Taygar, Linnéa Engman, Ida Flink, Suzanne Petersson, Katja Boersma, Lance M. McCracken, Laura Simons, Johan W.S. Vlaeyen, Rikard K. Wicksell

**Affiliations:** 1 Department of Clinical Neuroscience, 27106 Karolinska Institute, Stockholm, Sweden; 2 Pain Clinic, Capio St. Göran Hospital, Stockholm, Sweden; 3 Department of Psychiatry and Neuropsychology and Alzheimer Centrum Limburg, Mental Health and Neuroscience Research Institute, 5211 Maastricht University, Maastricht, Netherlands; 4 Methodology of Educational Sciences Research Group, Faculty of Psychology and Educational Sciences, 54514 KU Leuven, Leuven, Belgium; 5 School of Behavioural, Social and Legal Sciences, 6233 Örebro University, Örebro, Sweden; 6 Centre for Psychiatry Research, Department of Clinical Neuroscience, 97092 Karolinska Institute,Stockholm, Sweden; 7 Stockholm Health Care Services, Stockholm, Sweden; 8 Department of Medicine and Optometry, 4180 Linnaeus University, Kalmar, Sweden; 9 Division of Clinical Psychology, Department of Psychology, 8097 Uppsala University, Uppsala, Sweden; 10 Department of Anaesthesiology, Perioperative and Pain Medicine, 10624 Stanford University School of Medicine, Palo Alto, CA, USA; 11 Experimental Health Psychology, 5211 Maastricht University, Maastricht, the Netherlands; 12 Health Psychology Research Group, 54514 KU Leuven, Leuven, Belgium

**Keywords:** digital < general, chronic pain, single-case experimental study, linear mixed models, randomization test

## Abstract

**Background:**

Chronic pain affects up to 20% of the population and often results in significant distress, disability, and reduced quality of life. Despite compelling evidence for behavioural treatments, treatment access remains limited. To improve access, a digital behavioural treatment for chronic pain was developed as part of the multi-phase ‘DAHLIA’ project. The present study evaluates the effects of this newly developed digital behavioural treatment.

**Methods:**

Data were collected in a single-arm, iterative trial using a replicated single-case experimental design (SCED). In total, 56 participants received treatment (mean age 47, 87% female, varied pain regions). Participants completed twice daily digital diaries and questionnaires at pre-, and post-treatment, and at 3- and 6-months follow-ups. Diary items and questionnaires assessed psychological flexibility and acceptance, pain-related functioning, pain intensity, and general well-being. Daily diary data were examined using a combined p-value approach to conduct a meta-analysis of the SCED data, and questionnaire data were analysed using linear mixed models.

**Results:**

Meta-analyses of daily diary data showed significant improvements in most pain-related items, indicating the utility of treatment in everyday life. Linear mixed model analyses of effects showed significant improvements over time in key outcomes with the largest changes observed in pain acceptance and psychological flexibility and smaller effects in pain-related functioning.

**Conclusion:**

This study provides initial evidence for the newly developed digital behavioural treatment for chronic pain. Findings suggest that the treatment can improve resilience, daily functioning and symptoms and suggest the potential of the treatment as an effective evidence-based treatment for chronic pain.

## 1. Background

Chronic pain, defined as pain persisting or recurring more than three months,^1^ is a prevalent and heterogeneous condition^2^ that affects 20-30% of the population worldwide.^2,3^ Chronic pain commonly results in significant distress and disability for the individual, and a financial burden for the society.^4^ Traditionally, pain management has focused on symptom alleviation, with limited effects on health-related quality of life and daily functioning.^5^

Today, there is strong empirical support for behavioural approaches aimed at enhancing resilience,^6–8^ defined as the ability to overcome adversity while maintaining adaptive functioning.^9^ Such approaches are normally based on cognitive behavioural (CB) frameworks, including the fear-avoidance model^10^ and the psychological flexibility model.^11^ From a CB perspective, resilience can be targeted by increasing psychological flexibility (PF) and its core processes such as acceptance.^12^ PF is defined as the capacity to adapt behaviour while staying open to thoughts and feelings, using the situation constructively, and acting in line with one’s goals and values.^11^ Improvements in PF and acceptance are in turn associated with improved functioning and reduced disability in individuals with chronic pain.^13,14^

Despite the empirical evidence supporting behavioural approaches such as cognitive behavioural therapy (CBT) and Acceptance and Commitment Therapy (ACT), few individuals with chronic pain have access to such treatments.^15^ Digital solutions offer a shift in how healthcare is accessed and delivered,^16^ particularly through increasing access to evidence-based treatments.^17^ The DAHLIA (Digital behaviourAl HeaLth for chronIc pAin) project intends to enhance healthcare standards by bridging the gap between empirical evidence and clinical practices.^18^ Within the DAHLIA program, a digital behavioural treatment for individuals with chronic pain was developed and iteratively refined,^19^ in accordance with the Medical Research Council’s framework for developing and evaluating complex interventions.^20^

Traditionally, clinical trials evaluating novel treatments for chronic pain have utilized standard comparisons between pre- and post-treatment assessments to determine treatment effects.^6–8^ However, there are several arguments why such an approach may not precisely capture treatment effects. Single-point pre- and post-treatment assessments may obscure important day-to-day fluctuations in symptoms, level of resilience and functioning. Also, natural fluctuations^21,22^ may appear during the treatment period and lead to an over- or underestimation of effects. Additionally, recall bias can affect the accuracy of retrospective questionnaires.^23^ To provide more reliable and more ecologically valid results, daily diary methods offer a complementary approach by capturing day-to-day variations in key target variables.^24^ Thus, daily diary methods allow researchers to reliably assess pain-related outcomes.^25^ This is particularly relevant for important constructs in chronic pain such as psychological flexibility and acceptance,^11^ which are expected to fluctuate over time^26^ and thus may be insufficiently captured by retrospective, group-level assessments.

Accordingly, current evidence highlights the need for approaches that capture within-person changes in psychological processes over time during treatment. Thus, the DAHLIA program employs a precision health approach^27^ with a focus on individual effects and variations and therefore utilizes a replicated randomized single case experimental design (SCED). In SCEDs, each participant is assessed frequently across different phases, including baseline and treatment, and serves as their own control, with a randomized timing of the treatment start.^28^

As part of the DAHLIA program, the present study aims to provide initial evidence of effects of a newly developed digital behavioural treatment for chronic pain in a sample of individuals with various chronic pain conditions. Building on previously published findings where preliminary evidence of the treatment’s potential benefits and the importance of using SCED to explore effects was provided,^29^ this study expands the analysis by including a larger cohort using data from the first three iterations of the optimization phase.^18^ In this study, effects are assessed by examining day-to-day and pre-post-follow-up data. In addition, data from the present study is used to evaluate if the daily diary items are sensitive to change in a digital behavioural treatment. Results from the present study will guide the design and methods used in a larger clinical trial with a randomized controlled design.

More specifically, this study aims to address the following research questions:

1. To what extent do daily assessments of psychological flexibility and acceptance, pain-related functioning, pain intensity and well-being change over the course of a digital behavioural treatment, in this heterogeneous sample consisting of individuals with distinctly different chronic pain conditions?
2. How do psychological flexibility and acceptance, pain-related functioning, pain intensity and well-being change from pre-treatment to post-treatment and follow-up among individuals with chronic pain receiving a digital behavioural treatment?

## 2. Methods

### 2.1. Study design

This replicated randomized multiple-baseline SCED study used pre- and post-treatment and follow-up assessments, along with digital diary data collected during the optimization phase of the multi-phase DAHLIA project.^18^ The optimization phase included several iterations with small cohorts to evaluate and improve the treatment. Iteration one preceded iteration two and three (region Kalmar and Stockholm), whereas iterations two and three were executed in parallel (iteration two: Stockholm, iteration three: region Kalmar). To evaluate the effects, a sequentially replicated SCED was used, as outlined in the previously published study protocol.^18^ Thus, the study examined individual-level treatment effects in a heterogenous chronic pain sample, consisting of individuals with various chronic pain conditions (see detailed description and results of iteration one^29^). In the present study, we examined group-level effects across three iterations.

### 2.2. Participants and recruitment

Minor adjustments were made across iterations to improve the recruitment process and enhance inclusivity (for example age, outreach). Participants’ inclusion criteria were: (i) age 18 or older, (ii) pain for at least three months, (iii) ability to communicate fluently in Swedish, and (iv) access to a computer/tablet and smartphone with internet connection. During iteration one, we also applied an upper age limit of 65 years. Exclusion criteria were: (i) severe psychiatric comorbidity (such as psychosis, suicidality), (ii) severe injury/illness requiring immediate treatment or examination, or expected to worsen in the next six months, and (iii) ongoing or recent behavioural treatment for chronic pain (<6 months during iteration one; <3 months during iteration two and three).

Clinical coordinators (LE and SP) provided information about the study to staff and managers in healthcare units at primary- and specialist care levels. Patients were informed about the study by their healthcare provider: psychologist, medical doctor, occupational therapist, or physiotherapist. In iterations two and three, we also posted flyers with information about the study and QR-codes such as in university campuses, gyms and subway stations.

Sample size was primarily based on SCED methodology, where inference relies on replication across independent cases.^30^ Methodological standards recommend a minimum of three demonstrations of effects.^31^ However, simulation studies using randomization tests (see section 2.6.1) suggest that adequate statistical power may require larger samples, with a minimum of four participants and 20 observations per participant.^32^ In addition, the project protocol pre-specified a target of 10 participants per to ensure a sufficient replication.^18^

### 2.3. Procedure

Therapists, consisting of licenced psychologists and psychotherapists, and master students in psychology under supervision, conducted eligibility screening via phone or video, following a semi-structured interview guide with inclusion and exclusion criteria. All participants provided written informed consent digitally via the secure web-based data collection tool Research Electronic Data Capture (REDCap^33^) prior to the initiation of the study. Consent was obtained by actively ticking electronic checkboxes for participation and data handling consent, and by entering their name and contact information. The informed consent sheet provided information on data collection and handling, the voluntary nature of participation, and the right to withdraw at any point without providing a reason.

After signing informed consent, participants completed baseline assessments, including socio-demographic information, and questionnaires (see section 2.5.2), and initiated the daily digital diary (see section 2.5.1). Subsequently, therapists provided access to the digital treatment (see section 2.4). Participants were instructed to complete questionnaires after treatment completion and at 3- and 6-months follow-ups after they completed treatment. In addition to twice daily during treatment, digital diary was also completed for 5 days at post-treatment and at 3- and 6-month follow-ups. No financial compensation was provided for participation.

In addition, weekly evaluations and exit interviews (see the protocol^18^ for details) were collected to assess feasibility, and will be presented elsewhere (Taygar et al., in preparation).

### 2.4. Treatment

The DAHLIA treatment (Swedish: ‘Leva med smärta’) was provided using the Swedish digital healthcare platform “1177 stöd och behandling” and consists of six-modules, and weekly digital therapist interaction following module completion. Each model consists of four short 15-minute self-guided micro-sessions of structured treatment content including psychoeducation reading materials and reflective writing exercises, designed to facilitate the application of learned skills and strategies in the participant’s daily life. Participants were able to choose the format of the weekly digital therapist interaction: video call, phone call or asynchronous chat. However, during iteration three, as part of the feasibility testing, participants were only able to chat with their therapist.

The treatment is based on learning theory and an integration of the fear-avoidance^10^ and psychological flexibility models.^11^ Treatment development utilized a user-centred approach during Phase 0 (preparation) and Phase 1 (design) of the DAHLIA project, including focus groups and iterative prototype testing. A detailed description of the process can be found in the development paper.^19^ Minor adjustments were made to the treatment throughout the optimization phase (between iterations), while the structure and content overview remained consistent across all iterations. Booster sessions were offered after two and four months to help maintain treatment effects. Table 1 provides an overview of the treatment content and structure (also presented in a previously published paper for iteration one^29^).

**Table 1.** Overview of treatment content.

Module	Session	Content of the micro-sessions
1 – You and your pain	1	Psychoeducation on recurring pain and psychological treatment of pain
	2	Psychoeducation on the interplay between psychological factors and pain
	3	The role of behaviour in connection to pain and vicious circles
	4	Functional analysis of behaviour in relation to long- and short-term consequences
2 – Where do you want to go?	1	Introduction to values in different areas of life
	2	Life values in relation to family and close relationships
	3	Values in relation to friends and health
	4	Values in relation to work/education and leisure activities
3 – What do you want to achieve?	1	Values in relation to goals and goals in relation to family and close relationships
	2	Goals in relation to friends and health
	3	Goals in relation to work/education and leisure activities
	4	Reflections on a life led in the direction of one’s values
4 – Moving forward	1	Taking steps towards goals based on values
	2	Taking difficult steps towards goals even when it is difficult
	3	Negative thoughts and emotions as part of change and strategies to handle catastrophic thoughts
	4	Using the support from others
5 – Increasing strength and resilience	1	Building skills to handle difficult change – i.e. decreasing avoidance behaviour through exposure
	2	Dissecting goals and steps into manageable parts
	3	Emotion regulation strategies for strong affects
	4	Acceptance and validating strategies for self-criticism
6 – The road ahead	1	Creating detailed plans of steps to take in relation to goals
	2	Repeating actions to form new habits
	3	Setting up plan for maintaining progress after the treatment
	4	Reflections on treatment progress and planning for the future

Across iterations, a total of ten therapists provided treatment (n=8 female, n=2 male; aged 24-58 years; 0-33 years of clinical experience and 0-30 years of experience working with chronic pain patients). Clinical coordinators briefed therapists on how to deliver the treatment and instructed them to follow the treatment manual. Furthermore, in Kalmar, therapists who were not affiliated with the research team participated in check-in meetings convened four times per semester and were provided access to support as required. During these check-in meetings, therapists could ask questions about the treatment delivery and discuss potential challenges encountered in practice.

### 2.5. Data collection

#### 2.5.1. Digital diary

Participants completed a digital diary using the m-Path smartphone application.^34^ Diary items were selected following established outcome recommendations in chronic pain research, the IMMPACT (Initiative on Methods, Measurement, and Pain Assessment in Clinical Trials) recommendations.^35^ Also, the constructs measured using questionnaires are consistent with the goals of optimization frameworks such as MOST (The Multiphase Optimization Strategy; refs. 36), and principles of process-based therapy.^37^ Thus, the digital diary assessed (i) process outcomes: psychological flexibility and acceptance (including avoidance and engagement in meaningful activities), (ii) primary outcomes: pain-related functioning (such as self-efficacy, catastrophizing, interference), and (iii) secondary outcomes: pain intensity and general well-being (including pain intensity, sleep, mood, fatigue, stress, social support).

Diary items were piloted in a single-case observation design (SCOD) study,^38^ and discussed with the research team, experts in the experience sampling methods, and healthcare professionals. Diary items proposed in the study protocol^18^ were refined accordingly. Items were rated on a 7-point Likert scale following the design of Experience Sampling trials.^39^ Notable exceptions are pain intensity which was measured on a 0-10-point scale following the Numeric Rating Scale for Pain^40^, and a multiple-choice item assessing pain interference. See Table 2 for daily diary items (also presented in a previously published paper on iteration 1^29^).

**Table 2.** Diary items used in the present study.

​	Construct	Question	Answer scale
Process outcomes	Pain avoidance	*During the morning/afternoon,*… I stopped doing things because of my pain.	7-point numeric scale (1: not at all – 7: very much)
	Engagement in meaningful activity	… I did things that are important to me.	
	General avoidance	… I avoided doing things because of how I felt.	
Primary outcomes	Pain self-efficacy	… I felt confident that I could do activities despite my pain.	7-point numeric scale (1: not at all – 7: very much)
	Pain catastrophizing	… I worried about my pain getting worse or not.	
	Pain interference*	… my pain interfered with …	Multiple-choice:
			- General activities
			- Mood
			- Walking abilities
			- Normal work (including housework)
			- Relationship with others
			- Enjoyment of life
			- Did not disturb
Secondary outcomes	Pain intensity	... my pain level was: 1, 2, 3, 4, 5, 6, 7, 8, 9, 10	10-point numeric scale (1: no pain – 10: worse possible pain)
	Positive affect	…I felt happy, energetic, or enthusiastic.	7-point numeric scale (1: not at all – 7: very much)
	Negative affect	… I felt sad, irritated, depressed, or hopeless.	
	Sleep (General)^1^	Last night, I generally slept well.	
	Sleep (Falling asleep)^1^	Last night, I had problems falling asleep.	
	Sleep (Waking up)^1^	Last night, I woke up frequently or too early.	
	Stress	*During the morning/afternoon,*… I felt stressed.	
	Fatigue	… I felt tired.	
	Social support	… I felt supported by others.	

^1^Only included in the lunch questionnaire.

*Not included in the analysis as it yielded nominal data.

*Note.* In the study, the items were presented in Swedish and translated into English for transparency.

Participants were prompted to complete the digital diary twice daily (at 12.00 and 18.00) prior, during, and after the treatment. Diary entries were open for two hours and a reminder was sent after one hour if not completed. Participants were requested to complete the digital diary for a randomized period of 5-10 days prior to the treatment (baseline, or A-phase). In iterations two and three, the baseline length was extended to 7-10 days to attempt to achieve higher baseline stability. The exact baseline length for each participant was determined through randomization, and the diary was scheduled to align with each participant’s treatment start date as scheduled by the treating therapist. During the treatment phase (B-phase), diaries were initially scheduled for six weeks (treatment), plus 5 days following end of treatment (post-treatment), with an option to extend the daily diary if participants engaged with the treatment for a longer period.

#### 2.5.2. Questionnaires

See Table 3 for the list of questionnaires (also presented in a previously published paper on iteration 1^29^; reduced number of instruments compared to the protocol^18^ to reduce respondent burden). As with daily diary items, questionnaires were selected following established outcome recommendations in chronic pain research,^35^ and followed the goals of optimization frameworks,^36^ and principles of process-based therapy.^37^ Thus, questionnaires were also selected to assess (i) process variables (psychological flexibility and acceptance), (ii) primary outcomes: pain-related functioning (including self-efficacy and catastrophizing), and (iii) secondary outcomes: (pain intensity and general well-being, specifically health-related quality of life, perceived stress, insomnia, anxiety, and depression,).

**Table 3.** Questionnaires used in the present study.

Focus	Variable	Instrument
Process outcomes	Pain Acceptance	Chronic Pain Acceptance Questionnaire (CPAQ-8)^41^
	Psychological flexibility	Multidimensional Psychological Flexibility Inventory (MPFI-24)^42^
	Avoidance/Fusion	Psychological Inflexibility in Pain Scale (PIPS)^43^
Primary outcomes: Pain-related functioning	Catastrophizing	Symptom Catastrophizing Scale (SCS)^44^
	Pain self-efficacy	Pain Self-Efficacy Questionnaire (PSEQ)^45^
	Work ability	Work Ability Index (WAI)^46^
	Functioning	Brief Pain Inventory – Short Form (BPI-SF)^47^
Secondary outcomes: General well-being	Health-related quality of Life	EuroQol 5-Dimension Questionnaire (EQ-5D)^48^
	Perceived stress	Perceived Stress Scale (PSS)^49^
	Anxiety	Generalized Anxiety Disorder Assessment (GAD-7). 5-Dimension Questionnaire.^50^
	Depression	Patient Health Questionnaire (PhQ-9)^51^
	Insomnia	Insomnia Severity Index (ISI)^52^

### 2.6. Analyses

Data were downloaded from REDCap and m-Path. Python scripts were used to add NA (Not Available) markers to missing diary data, conduct statistical analyses of daily diary, and calculate individual questionnaire scores. An alpha level of .05 was used as criteria for statistical significance. As a strategy to address multiple comparisons, we applied the Benjamini–Hochberg procedure to control for false discovery rate (FDR) at 5%^53^ when analysing both daily diary and questionnaires.

#### 2.6.1. Aggregation of individual-level effects using daily assessments

Aggregated statistical significance tests for all iterations were conducted using the SCED data from the daily diaries. We excluded participants with very low diary compliance rates (<30%), or little data collected during either phase (<5 data points) following the design of similar studies^54^ and established guidelines.^30^ We also excluded participants who dropped out before starting module 2, as we did not expect changes to occur that early in treatment.

To assess changes in daily diary measures, we first applied an AB-phase Randomization test (RT) for each participant and item, adjusting for gradual and delayed effects.^55^ The RT compared the observed series to an ideal gradual response function (ramp) remaining at zero during the A-phase and increasing monotonically (for example 1-2-3) during the B-phase. The ramp was constant during the baseline phase and then increased linearly throughout the treatment, allowing to measure gradual effects.^55^ To account for delayed treatment effects, we set a delay of 1/6^th^ of the data after the treatment onset. This delay allows for detection of delayed gradual effects following the first contact with the therapist (occurring after completion of the first module), as we expect changes to begin to occur following the first module. For drop-out participants, we applied a delay which was proportional to the completed portion of the treatment, hence, 1/3 of the B-phase if the participant completed module 3.

To evaluate group-level treatment effects measured with diary items, we used a combined p-value approach.^56^ Specifically, we summed individual directional p-values across participants for each diary item, which we then compared to a reference distribution of sums generated under the null hypothesis with the p-values following a uniform [0,1] distribution.

#### 2.6.2. Determining pre-post-follow-up treatment effects

To evaluate changes over time, a priori specified analyses were conducted using linear mixed model analyses for each questionnaire using Jamovi.^57^ Assumptions of normality for difference scores were evaluated using the Shapiro–Wilk test and Q–Q plots. Effect sizes (Cohen’s d) were calculated for each variable.

## 3. Results

### 3.1. Participant flow and characteristics

**Figure 1.**
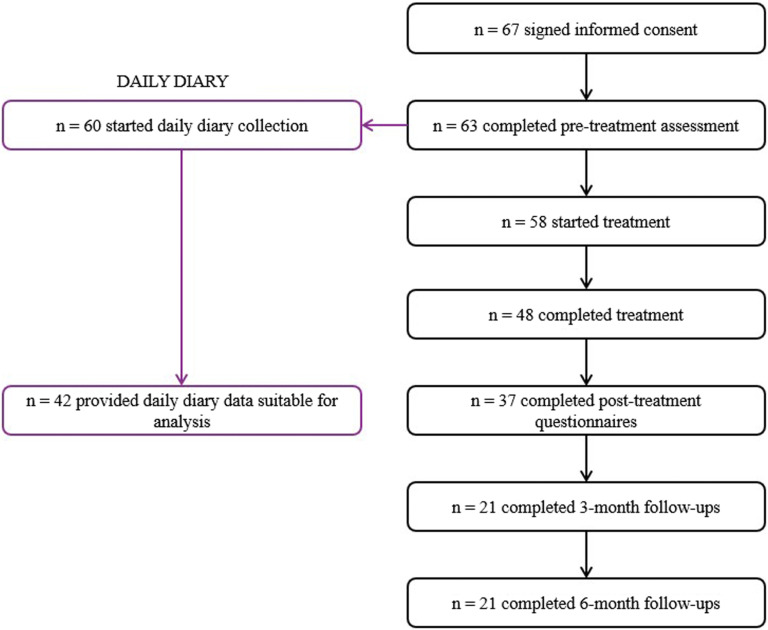
Participant flow through the study. *Note.* All participants who completed baseline assessments were included in the pre-post-follow-up analysis.

Figure 1 provides a flow chart for the study. Across all iterations, n=58 participants started the treatment. Number of completers and dropout rates varied by iteration; in iteration one, n=9 participants competed the treatment and n=2 dropped out (18%), in iteration two, n=28 participants completed the treatment and n=4 dropped out (14%), and in iteration three, n=11 participants completed the treatment and n=4 dropped out (26%).

**Figure 2.**
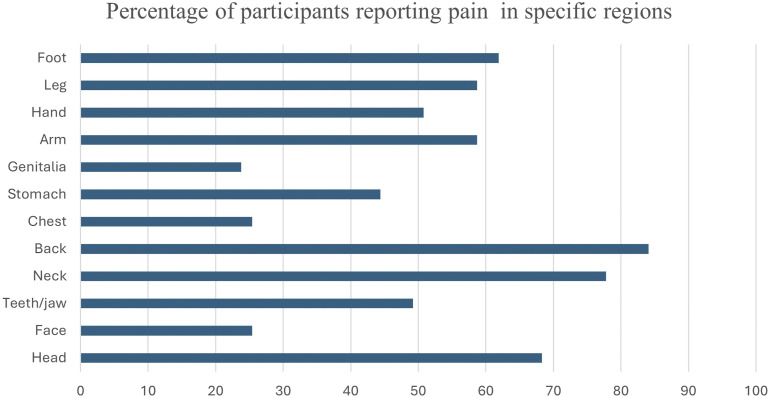
Percentage of participants reporting pain in specific regions. *Note.* Percentages include participants reporting widespread pain.

Participants were predominately women (87%), with a mean age of 47 years (SD = 14.28, range = 21–83). Half of the participants (50,8%) reported widespread pain. Most participants reported having experienced chronic pain for several years. Reported pain duration ranged from approximately 3 months to 40 years; however, several participants described their pain duration in non-specific terms (“several years” or “many years”), precluding precise calculation of summary statistics. Frequency of pain in specific body regions are presented in Figure 2.

**Figure 3.**
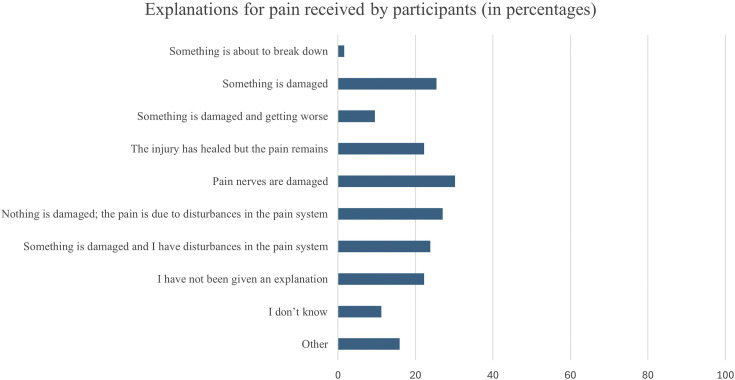
Explanations for pain received by participants (in percentages). *Note.* Multiple responses allowed.

Most participants reported having received an explanation for their pain, as reported in Figure 3 below.

### 3.1. Daily diary

Table 4 shows an overview of the effects in the daily diary measures based on comparisons between the baseline phase (5–10 days) and the treatment phase (6–8 weeks). In short, significant improvements were seen in the key outcome variables with consistent improvements in pain avoidance, pain self-efficacy, and engagement in meaningful activities. In contrast, some items, such as pain intensity and pain catastrophizing, showed no consistent change.

**Table 4.** Combined p-values of daily diary measures by construct (meta-analysis of randomization tests).

​	Construct	Combined p-value
Process outcomes	Pain avoidance	0.021*
	Engagement in meaningful activity	0.010*
	General avoidance	0.024*
Primary outcomes	Pain self-efficacy	0.010*
	Pain catastrophizing	0.080
Secondary outcomes	Pain intensity	0.141
	Positive affects	<0.001*
	Negative affects	0.002*
	Sleep (General)	0.004*
	Sleep (Falling asleep)	0.335
	Sleep (Waking up)	0.150
	Stress	0.764
	Fatigue	0.113
	Social support	0.227

*Note. *Diary item pain interference was not included in the analysis as the multiple-choice format does not allow for gradual comparisons.

*Significant using Benjamini-Hochberg adjustment α for multiple comparisons; raw p-values are reported.

### 3.2. Pre-post-follow-up questionnaires

**Figure 4.**
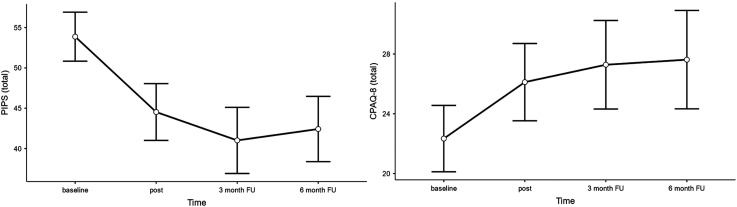
Plots depicting predicted means across time points in process outcome measures psychological inflexibility in pain (PIPS) and pain acceptance (CPAQ-8).

Results of the linear mixed model analyses are presented in Table 5. Assumption checks for the models were conducted and considered acceptable. As shown in Figure 4, participants showed significant and maintained improvements in process outcomes, with the largest changes observed in activity engagement (CPAQ-8 subscale ‘Engagement in meaningful activities’: post: Δ = 2.24, p < .001, d = 0.90; 3-month follow-up: Δ = 2.63, p < .001, d = 0.86; 6-month follow-up: Δ = 3.18, p < .001, d = 0.86) and pain avoidance (PIPS subscale ‘Pain avoidance’: post: Δ = -7.73, p < .001, d = -1.50; 3-month: Δ = -9.79, p < .001, d = -1.53; 6-month: Δ = -8.94, p < .001, d = -1.42). No significant changes were observed for psychological flexibility according to the Multidimensional Psychological Flexibility Inventory (MPFI-24).

**Table 5.** Estimated marginal means and standard errors in outcome measures by time point (linear mixed model analysis).

	Outcome	Baseline	ΔPost (SE)	P-value	Cohen’s d	Δ3FU	P-value	Cohen’s d	Δ6FU	P-value	Cohen’s d
Process outcomes	CPAQ-8 (total)	25,84	3,78 (1,04)	<,001*	0,87^a^	4,94 (1,27)	<,001*	0,93^a^	5,28 (1,46)	<,001*	0,87^a^
	Engagement in Activity	14,19	2,24 (0,60)	<,001*	0,90^a^	2,63 (0,74)	<,001*	0,86^a^	3,18 (0,85)	<,001*	0,86^a^
	Willingness to Have Pain	11,70	1,61 (0,61)	0,01*	0,62^b^	2,37 (0,75)	0,00*	0,74^b^	2,17 (0,87)	0,01*	0,74^b^
	MPFI										
	Flexibility	4,2	0,278 (0,11)	0,016*	0,56^b^	0,41 (0,14)	0,00*	0,67^b^	0,68 (0,16)	<,001*	0,98^a^
	Inflexibility	2,9757	-0,082 (0,08)	0,306	-0,24	-0,09 (0,10)	0,34	-0,23	-0,16 (0,11)	0,154	-0,23
	PIPS (total)	45,46	-9,34 (1,45)	<,001*	-1,44^a^	-12,86 (1,80)	<,001*	-1,60^a^	-11,45 (1,77)	<,001*	-1,60^a^
	Avoidance	27,64	-7,73 (1,16)	<,001*	-1,50^a^	-9,79 (1,45)	<,001*	-1,53^a^	-8,94 (1,42)	<,001*	-1,42^a^
	Fusion	17,81	-1,62 (0,55)	0,004*	-0,65^b^	-3,07 (0,68)	<,001*	-0,99^a^	-2,53 (0,67)	<,001*	-0,83^a^
Primary outcomes	SCS	6,806	-0,99 (0,35)	0,006*	-0,64^b^	-1,93 (0,43)	<,001*	-1,00^a^	-1,79 (0,42)	<,001*	-0,94^a^
	PSEQ	8,15	1,29 (0,42)	0,003*	0,67^b^	1,43 (0,51)	0,01*	0,61^b^	1,56 (0,51)	0,003*	0,67^b^
	WAI	25,4	-1,18 (0,90)	0,194	-0,31	-1,17 (1,15)	0,31	-0,24	-2,91 (1,32)	0,031*	-0,53^b^
	BPI-SF										
	Pain severity	4,71	-1,32 (0,35)	<,001*	-0,81^a^	-1,57 (0,44)	<,001*	-0,78^b^	-0,96 (0,43)	0,03*	-0,49^c^
	Pain interference	4,86	-0,46 (0,23)	0,05	-0,44	0,00 (0,29)	0,99	0,00	-0,34 (0,28)	0,23	-0,26
Secondary outcomes	EQ5D	0,8365	0,038 (0,01)	0,006*	0,67^b^	0,04 (0,02)	0,01*	0,63^b^	0,02 (0,02)	0,196	0,31
	PSS10	15,59	-2,60 (0,98)	0,01*	-0,61^b^	-4,35 (1,20)	<,001*	-0,83^a^	-3,80 (1,39)	0,01*	-0,64^b^
	GAD7	6,01	-1,33 (0,679)	0,054	-0,47	-3,05 (0,83)	<,001*	-0,87^a^	-2,27 (0,96)	0,021*	-0,57^b^
	ISI	14,181	-0,734 (0,89)	0,411	-0,19	-1,86 (1,09)	0,09	-0,40	-0,69 (1,25)	0,586	-0,13
	PHQ9	10,22	-1,5 (0,79)	0,062	-0,45	-2,09 (0,97)	0,03*	-0,51^b^	-1,32 (1,11)	0,239	-0,28

*Note.* All participants who completed baseline assessments (n=63) were included in the analysis.

*Significant using Benjamini-Hochberg adjustment α for multiple comparisons; raw p-values are reported.

ΔPost = estimated change from baseline to post-treatment; Δ3FU = estimated change from baseline to 3-month follow-up; Δ6FU = estimated change from baseline to 6-month follow-up.

^a^ = large (|d| ≥ 0.8),

^b^ = moderate (|d| = 0.5–0.79),

^c^ = small (|d| = 0.2–0.49).

PIPS: Psychological Inflexibility in Pain Scale. CPAQ-8: Chronic Pain Acceptance Questionnaire. MPFI-24: Multidimensional Psychological Flexibility Inventory-24. PSEQ: Pain Self-Efficacy Questionnaire. SCS: Symptom Catastrophizing Scale. BSI-SF: Brief Pain Inventory – Short Form. WAI: Work Ability Index. PHQ-9: Patient Health Questionnaire-9. GAD-7: Generalized Anxiety Disorder Assessment. PSS: Perceived Stress Scale. ISI: Insomnia Severity Index. EQ5D: EuroQol 5-Dimension Questionnaire.

**Figure 5.**
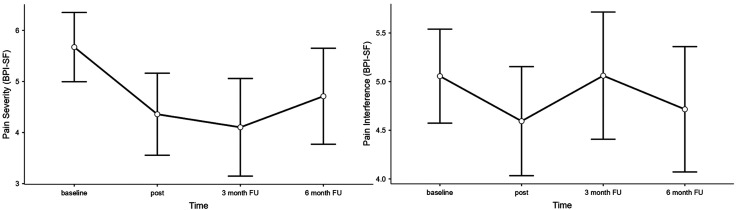
Plots depicting predicted means across time points in the primary outcome measure pain severity and pain interference.

Several improvements were observed for the primary outcomes. As seen in Figure 5, pain severity (BPI-SF subscale ‘Pain Severity’) showed large improvements at pre to post (Δ = -1.32, p < .001, d = -0.81), and small to medium changes at follow-ups (3-month follow-up: Δ = -1.57, p < .001, d = -0.78; 6-month follow-up: Δ = -0.96, p = .03, d = -0.49) whereas pain interference (BPI-SF subscale ‘Pain Interference’) showed no significant changes. Secondary outcomes improved mainly at follow-ups in stress (PSS-10), anxiety (GAD-7), and quality of life (EQ5D).

## 4. Discussion

This study evaluated the effects of a digital behavioural treatment designed to enhance resilience and daily functioning in individuals with chronic pain. We analysed data from three iterations completed during the optimisation phase of the multi-phase DAHLIA project.^18^ The primary objective was to examine initial evidence of treatment effects on psychological flexibility and acceptance, indicators of pain-related functioning, and secondary outcomes including pain intensity and general well-being. These findings are intended to inform the design of a larger randomized controlled trial enhanced with single-case experimental methodology.

### 4.1. Benefits of digital behavioural treatments for chronic pain

Findings suggest improvements in psychological flexibility, acceptance, and pain-related functioning, as demonstrated in both daily diary data and pre– to follow-up assessments of questionnaire data. Overall, daily diary data and questionnaires generated similar results (with minor inconsistencies, see Section 4.2), providing complementary support for the treatment’s ability to improve resilience and functioning in people with chronic pain. Participants showed increased pain acceptance, greater psychological flexibility, and higher engagement in meaningful activities, alongside reductions in pain avoidance. Notably, findings were mixed for pain intensity: while questionnaire data (pre– to follow-up) showed significant improvements, daily diary data did not illustrate corresponding effects. Furthermore, general well-being outcomes suggested improved mood, while results on sleep measures were inconsistent.

These findings align with previous research showing strong empirical evidence for behavioural treatments for chronic pain, where improvements are more consistently observed in pain-related functioning and psychological processes (i.e. pain catastrophizing, pain acceptance, psychological inflexibility) rather than in pain intensity itself.^6–8^ Additionally, the results correspond with the growing body of research supporting digital delivery formats of behavioural interventions targeting chronic pain.^58,59^ The observed improvements also support the treatment’s proposed theoretical mechanisms, aligning with both the fear-avoidance model^10^ and the psychological flexibility model.^11^ As such, empowering individuals with chronic pain to respond to daily stressors using high psychological flexibility instead of an avoidance and catastrophizing mindset can improve functioning and well-being in this population.^13,60^

Moreover, the use of daily diary data contributes to the growing literature emphasizing high-frequency, ecologically valid measurement to capture dynamic treatment effects.^24,25^ By showing both consistencies and inconsistencies between questionnaires and daily diary data (see section 4.2), this study emphasizes how different types of measures may capture distinct aspects of change. As seen in previous research, information about how the pain manifests in daily life may influence how patients perceive their situation and how it can be managed.^61^

The lower attrition rate in the third iteration suggests a need for flexibility and person-centred support in treatment delivery. Tentatively, a slightly higher rate of dropouts in iteration three compared to iterations one and two may relate to restricting the patient-therapist communication to asynchronous chat-based contact. This observation corresponds to participant reports during the development phase, highlighting the value of flexibility in the communication with therapists.^19^ The feasibility and features of the treatment will further be explored in a feasibility trial.^18^

### 4.2. Methodological considerations

Of methodological relevance, inconsistent results were seen within the same measurement domains. Psychological inflexibility measured by the subscale of the Multidimensional Psychological Flexibility Inventory-24 (MPFI-24) did not show any significant changes despite medium to strong effects observed in psychological inflexibility in Pain (Psychological Inflexibility in Pain Scale (PIPS)) and pain acceptance (Chronic Pain Acceptance Questionnaire (CPAQ-8)). This difference may be due to PIPS and CPAQ-8 assessing pain-specific psychological flexibility and acceptance,^43,62^ whereas MPFI-24 measures broader psychological flexibility and inflexibility^63^ not affected by the treatment.

Moreover, while significant improvements were seen in measures of psychological inflexibility in pain (PIPS) and pain acceptance (CPAQ-8), as well as in daily diary measures of pain avoidance and engagement in meaningful in activities, changes in pain interference measured by the BPI-SF interference subscale were non-significant. Importantly, prior research shows that changes in pain interference are closely associated with changes in psychological inflexibility in pain and pain acceptance,^64^ suggesting present null findings in overall pain interference may not necessarily indicate the absence of treatment-related change. This discrepancy may however reflect the broad range of domains covered by the BPI-SF interference subscale, with one item per domain (general activity, mood, walking, relationships, sleep, enjoyment of life).^65^ Possibly, improvements in pain interference occurred in specific domains, with a lower overall responsivity of the total interference score, as noted in prior research.^66^ Future studies could consider other items measuring pain interference such as the Pain Interference Index^67^ and Pain Disability Index.^68^

Furthermore, no changes were observed for the daily diary item pain catastrophizing despite clear improvements in pain-related functioning captured both by questionnaires and daily diary items, and improvements in symptom catastrophizing captured by the Symptom Catastrophizing Scale (SCS). This difference may reflect limitations with using a single-item measurement, as pain catastrophizing is increasingly understood as a multifaceted construct.^69^ Although single-item measures can have good validity, they might not always be adequate when measuring multifaceted constructs,^70^ suggesting that a multiple-item diary approach might be more appropriate for such constructs.

In the pain severity outcomes, significant improvements were seen in the pre-to follow-up questionnaire data but not in the daily diary ratings. This finding could be due to the high variability in reporting of pain intensity^71^ and the natural fluctuations of symptoms.^21,22^ As a reliable measure of pain severity requires multiple measurement points across several days,^72^single-point questionnaires might be prone to random error and therefore be less reliable as measures of pain levels in the chronic pain population. Accordingly, retrospective questionnaires may capture global, evaluative judgments of pain. As pain experience is typically influenced by psychological and social factors,^73^ retrospective questionnaires might be more affected by improvements in overall well-being. Daily diary measures, however, reflect momentary pain experiences and are less influenced by susceptible to recall bias and retrospective reconstruction^23,74^ and may therefore be less influenced by overall improvements.

Additionally, no significant improvements were observed regarding sleep according to the Insomnia Severity Index (ISI), although daily diary items showed improvements in general sleep quality. ISI measures several constructs, including falling asleep, staying asleep, waking up early, satisfaction, interference, notice ability, and distress,^75^ while single daily diary items, on the other hand, specifically assess single constructs (such as sleep satisfaction). Similar discrepancies have previously been reported in other studies, as ISI shows moderate to poor convergent validity with sleep diary parameters.^76^

Thus, inconsistent effects across measures of the same or similar domains illustrate the importance of utilizing adequate instruments, and points at the relevance of evaluating the psychometric properties of daily diary items for chronic pain trials as well as further developing and updating guidelines.

### 4.3. Strengths, limitations and recommendations for future research

The strengths of this study include the use of a replicated SCED, as it allows for detailed, within-person evaluations^77^ across three treatment iterations, providing a strong basis for examining initial evidence of treatment effects. Also, the combination of pre-post-follow-up questionnaires with daily diary assessments offers a comprehensive view of general clinical improvements and more specific day-to-day changes in functioning, as each approach offers distinct advantages.^78^ Assessing multiple change processes, namely psychological flexibility, acceptance, pain-related functioning, pain intensity, and well-being, further allowed for a detailed discussion of key change mechanisms as well as methodological challenges.^35^ Additionally, testing the treatment continuously during the iterative development of the digital intervention allowed for data-driven decisions on how to improve both the treatment and the assessment, prior to evaluating it in a larger randomized controlled trial.^36,79^

Several limitations should be considered when interpreting the findings from the present study. The single-arm design and modest sample size limit causal inference and generalizability.^80^ Although control groups are not typically required in single-case experimental designs, as inference is based on repeated within-person comparisons across baseline and intervention phases comparing the individual to their own baseline as a control period,^28,30^ the absence of a concurrent control condition limits causal interpretation especially of the linear mixed model estimates. Observed pre–post-follow-up changes cannot fully exclude alternative explanations such as natural symptom fluctuation, regression to the mean, or expectancy effects.^80^ However, an RCT is currently being conducted within the DAHLIA project,^18^ allowing for within- and between-individual comparisons across allocations.

Moreover, the sample was predominantly female (87%) and the intervention was delivered exclusively in a digital format, which may limit generalizability to male patients and to populations with limited access to or familiarity with digital interventions. Also, in iteration one, an upper age limit of 65 years was applied as part of the inclusion criteria, primarily due to the initial focus on the working population. Although this criterion was only applied in the first iteration, only a small proportion of the sample were older adults (6%), limiting the representativeness of the sample for older adults.

In addition, the exclusion of participants with low diary compliance or early dropout may have introduced attrition bias,^80^ as individuals who remained in the analyses may differ systematically from those excluded, potentially leading to an overestimation of treatment effects. However, these criteria were applied to ensure sufficient data quality and valid estimation in the SCED analyses.^30,54^ Also, as mentioned, some assessments (such as pain catastrophizing) used single items, which may not fully capture their multidimensional nature,^70^ which may be reflected in discrepancies between questionnaire and diary outcomes. Moreover, the patient-therapist communication differed across iterations, with asynchronous text messaging in iteration three. Additionally, follow-up periods were not long enough to reliably evaluate long-term effects,^81^ which was considered outside the scope of the study. Furthermore, the low response rate at follow-up limits the interpretability of long-term effects. Also, treatment fidelity was not formally assessed, and it is therefore unclear to what extent the intervention was delivered consistently across therapists and iterations. However, the micro-session content was fully standardized and delivered in a fixed order across all participants, limiting variability in the core digital intervention component. Potential variation in fidelity is therefore primarily related to the adjunct therapist support rather than the intervention content itself. Also, most therapists were affiliated with the research group and involved in the development and evaluation of the intervention, which may have supported consistency in delivery. In addition, clinicians not affiliated with the research group were offered regular supervision and access to support when needed. Future studies should consider adding measures of treatment fidelity.

Hence, the study provides initial evidence of the treatment’s potential utility to increase resilience and daily functioning in patients with chronic pain, and future research should be conducted to validate these treatment effects. Based on the DAHLIA protocol for development and evaluation,^18^ a SCED-enhanced RCT may be conducted following treatment optimization, informed by findings that may suggest adjustments in, for instance, daily diary items and questionnaires. Furthermore, future studies may include analyses of cost-effectiveness and post-market surveillance as well as mediators and moderators of change, to provide more detailed insight regarding the benefits of the treatment. Also, future research should further investigate whether treatment effects differ across demographic or clinical subgroups. Additionally, future research may consider tailoring the treatment to subgroups with specific profiles or needs, such as pain after cancer, endometriosis, or comorbid insomnia.

### 4.4. Conclusion

The present study, with a replicated SCED approach to evaluate treatment effects in a chronic pain sample, provides initial evidence for the potential benefits of this novel digital behavioural treatment. The findings suggest the DAHLIA treatment generates meaningful improvements in key outcomes, including psychological flexibility, pain acceptance, and several aspects of pain-related functioning. These findings warrant a randomized controlled trial and add to the growing support for digital health treatments for individuals living with chronic pain.

## Data Availability

The data supporting the findings of this study is available upon reasonable request.*
